# SparseRNAfolD: optimized sparse RNA pseudoknot-free folding with dangle consideration

**DOI:** 10.1186/s13015-024-00256-4

**Published:** 2024-03-03

**Authors:** Mateo Gray, Sebastian Will, Hosna Jabbari

**Affiliations:** 1 Department of Biomedical Engineering, University of Alberta, Street, Edmonton, T6G2R3 AB Canada; 2 Department of Computer Science CNRS/LIX (UMR 7161), Institut Polytechnique de Paris, Street, Paris, 10587 France

**Keywords:** RNA, MFE, Secondary structure prediction, Dangle, Sparsification, Space complexity, Time complexity

## Abstract

**Motivation:**

Computational RNA secondary structure prediction by free energy minimization is indispensable for analyzing structural RNAs and their interactions. These methods find the structure with the minimum free energy (MFE) among exponentially many possible structures and have a restrictive time and space complexity ($O(n^3)$ time and $O(n^2)$ space for pseudoknot-free structures) for longer RNA sequences. Furthermore, accurate free energy calculations, including dangle contributions can be difficult and costly to implement, particularly when optimizing for time and space requirements.

**Results:**

Here we introduce a fast and efficient sparsified MFE pseudoknot-free structure prediction algorithm, SparseRNAFolD, that utilizes an accurate energy model that accounts for dangle contributions. While the sparsification technique was previously employed to improve the time and space complexity of a pseudoknot-free structure prediction method with a realistic energy model, SparseMFEFold, it was not extended to include dangle contributions due to the complexity of computation. This may come at the cost of prediction accuracy. In this work, we compare three different sparsified implementations for dangle contributions and provide pros and cons of each method. As well, we compare our algorithm to LinearFold, a linear time and space algorithm, where we find that in practice, SparseRNAFolD has lower memory consumption across all lengths of sequence and a faster time for lengths up to 1000 bases.

**Conclusion:**

Our SparseRNAFolD algorithm is an MFE-based algorithm that guarantees optimality of result and employs the most general energy model, including dangle contributions. We provide a basis for applying dangles to sparsified recursion in a pseudoknot-free model that has the potential to be extended to pseudoknots.

## Introduction

Non-coding RNAs play crucial roles in the cell, such as in transcription [1], translation [1, 2], splicing [3, 4], catalysis [1, 5] and regulating gene expression [1, 3, 6, 7]. Since RNA’s function heavily relies on its molecular structure, facilitated by hydrogen bonding both within and between molecules, predicting and comprehending the structure of RNA is a dynamic area of research. It is reasonable to assume (without further knowledge) that RNA forms the structure with the lowest free energy [8, 9]. This is the motivation for algorithms that aim to predict the RNA minimum free energy (MFE) structure from the pool of exponentially many structures it can form. Such methods employ a set of energy parameters for various loop types, called an energy model; to find the free energy of a structure, they add up the energy of its loops. While prediction accuracy of these methods depends on the quality of their energy models, these methods are applicable to novel RNAs with unknown families or functions and for the prediction of the structure of interacting molecules. The large time and space complexity of MFE-based methods ($O(n^3)$ time and $O(n^2)$ space where *n* is the length of the RNA), however, restricted their applications to small RNAs. The sparsification technique was recently utilized in existing MFE-based algorithms to reduce their time and/or space complexity [10–17] by removing redundant cases in the complexity-limiting steps of the dynamic programming algorithms. While the majority of these methods focused on simple energy models, some expanded sparsification techniques to more realistic energy models [15–17]. To the best of our knowledge, no existing method has yet incorporated dangles energy contributions into a sparsified prediction algorithm. Dangle energies refer to the free energy contributions of unpaired nucleotides that occur at the end of a stem-loop structure.

**Fig. 1:**
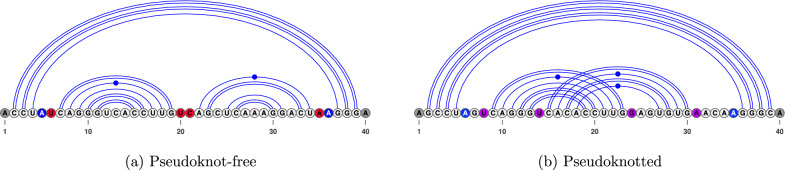
An RNA structure is shown with dangles highlighted. **a** In red, we have the dangles on the bands in the multi-loop. In blue, we have the dangle on the closing bases of the multi-loop. In gray, we have dangles on the outer end of the RNA. **b** We include purple to show dangles occurring in a pseudoknot. Dangles in pseudoknots can be handled differently depending on the program

We show in Fig. 1 the location of dangles on a pseudoknot-free structure (see Fig. 1a) and a pseudoknotted structure (see Fig. 1b). The complexity of dangles in a pseudoknot further increases as dangles have to be tracked for both bands within the pseudoknot.

Neglecting dangle energies in the prediction of RNA structure stability can lead to inaccuracies. For instance, a stem-loop structure that includes an unpaired nucleotide at the end may appear less stable than its actual stability if the dangle energy contribution is ignored. Conversely, a stem-loop structure with an unpaired nucleotide that interacts positively with another one may appear more stable than its actual stability if the dangle energy contribution is not taken into account.

Dangles, in some form, are implemented in the majority of MFE pseudoknot-free secondary structure prediction algorithms [18, 19]. RNAFold [18, 20–23, 25] is an $O(n^3)$ time and $O(n^2)$ space algorithm which implements the dangle 0 (“no dangle”), dangle 2 (“always dangle”), and dangle 1 (“exclusive dangle”) model (defined in Section “Dangles”). It also utilizes a dangle model that implements coaxial stacking—a type of stacking that gives a bonus to stacks in the vicinity of each other. LinearFold [19], a sparsified *O*(*n*) space heuristic algorithm has implemented the “no dangle” and “always dangle” model but has not implemented an “exclusive dangle” model. Fold from the RNAstructure library [26] is an $O(n^3)$ time and $O(n^2)$ space algorithm which implements an “exclusive dangle” model with coaxial stacking. MFold [27–29] is an $O(n^3)$ time and $O(n^2)$ space algorithm which has implemented an “exclusive dangle” model with coaxial stacking.

Handling dangles in pseudoknot prediction algorithms is less developed. Pknots [30], an $O(n^6)$ time and $O(n^4)$ space pseudoknot prediction algorithm has implemented an “exclusive dangle” model that also includes coaxial stacking. Within Pknots, a set of parameters is defined for pseudoknot-free and pseudoknot dangles. The pseudoknot parameters are estimated and rely on an estimated weighting parameter. Hotknots [31], a heuristic algorithm, uses the DP09 parameters, which include pseudoknotted parameters from Dirks and Pierce [32] and tuned by Andronescu et al. [31]; however, the energies for the pseudoknotted dangles are the same as those for pseudoknot-free dangles, and there is no weighting parameter.

### Contributions

In [15], we already discussed the sparsification of RNA secondary structure prediction by minimizing the energy in the Turner energy model. However, in this former work, we did not yet consider the energy contributions due to the interactions of base pairs at helix ends with dangling bases (i.e., ‘dangling ends’). Here, we identify the correct handling of dangling end energies in the context of sparsification as a non-trivial problem, characterize the issues, and present solutions.

For this purpose, we first state precisely how dangle energies are handled by energy minimization algorithms; to the best of our knowledge, this is elaborated here for the first time. Consequently, we devise novel MFE prediction algorithms that include dangling energy contributions *and* use sparsification techniques to significantly improve the time and space complexity of MFE prediction.

Like the algorithm in [15], our efficient SparseRNAFolD algorithm keeps the additional information to a minimum using garbage collection. In total, we study three different possible implementations and compare their properties, which make them suitable for different application scenarios. Finally, while we study the case of non-crossing structure prediction, we discuss extensions to the more complex cases of pseudoknot and RNA–RNA interaction prediction (such extensions being the main motivation for this work in the first place).

## Preliminaries: sparsification without dangling ends

We restate the preliminaries and main results from our former work on sparsification of free energy minimization without dangling ends [15].

We represent an *RNA sequence* of length *n* as a sequence $S=S_1,\dots ,S_n$ over the alphabet $\{A,C,G,U\}$; $S_{i,j}$ denotes the *subsequence* $S_i,\dots ,S_j$. A *base pair* of *S* is an ordered pair *i*.*j* with $1 \le i < j \le n$, such that *i*th and *j*th bases of *S* are complementary (i.e. $\{S_i,S_j\}$ is one of $\{A,U\}, \{C,G\},$ or $\{G,U\}$). A *secondary structure* *R* for *S* is a set of base pairs with at most one pairing per base (i.e. for all *i*.*j*, $i'.j'\in R$: $\{i,j\}\cap \{i',j'\}=\emptyset$). Base pairs of secondary structure *R* partition the unpaired bases of sequence *S* into *loops* [33] (i.e., hairpin loop, interior loop and multiloop). Hairpin loops have a minimum length of *m*; consequently, $j-i>m$ for all base pairs *i*.*j* of *R*. Two base pairs *i*.*j* and $i'.j'$ cross each other iff $i<i'<j<j'$ or $i'<i<j'<j$. A secondary structure *R* is *pseudoknot-free* if it does not contain *crossing base pairs*.

**Fig. 2:**
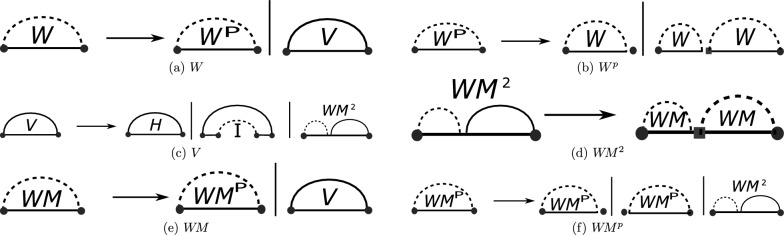
We show in graphical format each of the recursions. The notation for these figures is as follows: a solid horizontal line signifies the RNA sequence, a solid arc denotes a base pair, and dashed arcs represent regions. Fixed endpoints of a region are depicted by red circles, while blue squares indicate unpaired elements used for boundary determination

The unsparsified, original algorithm for energy minimization over pseudoknot-free secondary structures was stated by Zuker and Stiegler [24]. It is a dynamic programming algorithm that, given an RNA sequence *S* of length *n*, recursively calculates the minimum free energies (MFEs) for subsequences $S_{i,j}$ as $W(i,j)$ (stored in a dynamic programming matrix). Finally, $W(1,n)$ is the optimal free energy. We state this algorithm in a sparsification-friendly form following [15]. As usual, the algorithm is described by a set of recursion equations (for a minimum hairpin loop size of *m* and a maximum interior loop size of $M$)—see Fig. 2. For $1\le i<j\le n$, $i<j-m$:

1 $$\begin{aligned} W(i,j)&= \min \{\, W^p(i,j), V(i,j) \,\} \end{aligned},$$

2 $$\begin{aligned} W^p(i,j)&= \min \{\, W(i,j-1), \min _{i<k<j} W(i,k-1) + W(k,j) \,\} \end{aligned},$$

3 $$\begin{aligned} V(i,j)&= \min \{ \mathcal {H}(i,j); \hspace{-0.5em} \min _{\begin{array}{c} i<p<q<j\\ p-i+j-q-2\le M \end{array}} \hspace{-0.5em}\mathcal {I}(i,j;p,q) + V(p,q); W\!M^2(i+1,j-1)+a \} \end{aligned},$$

4 $$\begin{aligned} W\!M(i,j)&= \min \{\, W\!M^p(i,j), V(i,j) + b \,\} \end{aligned},$$

5 $$\begin{aligned} W\!M^p(i,j)&= \min \left\{\, W\!M(i+1,j) + c, W\!M(i,j-1) + c, W\!M^2(i,j) \,\right\}, \end{aligned}$$

6 $$\begin{aligned} W\!M^2(i,j)&= \min _{i<k<j} W\!M(i,k-1) + W\!M(k,j) \end{aligned}.$$

Here, *a*, *b*, *c* are multi-loop initialization penalty, branch penalty, and unpaired penalty in a multi-loop, respectively. $\mathcal {I}(i,j;p,q)$ refers to an interior loop between base pairs i.j and p.q. The initialization cases are $W(i,i)=0$; $V(i,j)=W\!M(i,j)=\infty$ for all $j-i\le m$ and $W\!M^2=\infty$ for all $j-i\le 2m+3$.

In these recursions, all function values (e.g. $W(i,j)$ or $W^p(i,j)$) denote minimum free energies over certain classes of structures of subsequences $S_{i,j}$. The classical Zuker/Stiegler matrices $W$, $V$ and $W\!M$ are defined as: $W$ yields the MFEs over general structures; $V$, over closed structures, which contain the base pair *i*.*j*; $W\!M$, over structures that are part of a multi-loop and contain at least one base pair.

Since sparsification is based on the idea that certain optimal structures can be decomposed into two optimal parts, while others (namely closed structures) are non-decomposable, we single out the partitioning cases and introduce additional function symbols $W^p$, $W\!M^p$, and $W\!M^2$.

### Recurrence visualization terminology

In Fig. 2, we visualize each of the recurrences listed in Eqs. (1) through 6. In our notation a solid horizontal line signifies the RNA sequence, a solid arc denotes a base pair, and dashed arcs represent regions. Fixed endpoints of a region are depicted by red circles, while blue squares indicate unpaired elements used for boundary determination.

### Sparsification without dangling ends

This allows us to cleanly explain the *key idea of sparsification* and consequently formalize it: to minimize over the energies of general structures in $W(i,j)$—note that there is another minimization inside of multi-loops that is handled analogously—the algorithm considers all closed structures *V*(*i*, *j*) and all others $W^p(i,j)$. Optimal structures in the latter class can be decomposed into two optimal structures of some prefix $S_{i,k-1}$ and suffix $S_{k,j}$ of the subsequence. Classically, the minimum is therefore obtained by minimizing over all ways to split the subsequence. Sparsification saves time and space since it is sufficient to consider only the splits where the optimum of the suffix $S_{k,j}$ is not further decomposable (formally, where $W(k,j) < W^p(k,j)$). Briefly (for more detail, see [15] or [10]), this is sufficient since otherwise there is a $k'$ to optimally split the suffix further into $S_{k,k'-1}$ and $S_{k',j}$. The split of $S_{i,j}$ at *k* cannot be better than the split at $k'$ and therefore does not have to be considered in the minimization; thus, it can be restricted to a set of *candidates*. This is argued by the *triangle inequality for* $W$ (which directly follows from the definition of $W$ as minimum):

$$\begin{aligned} W(i,j) \le W(i,k-1) + W(k,j) \qquad \text {for all}\,\, 1\le i< k\le j\le n. \end{aligned}.$$

**Fig. 3:**
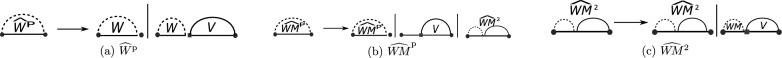
Graphical representation of the sparsified versions of the recursions: $W^p$, $W\!M^p$, and $W\!M^2$. The notation for these figures is as follows: a solid horizontal line signifies the RNA sequence, a solid arc denotes base pairs, and dashed arcs represent regions. Fixed endpoints of a region are depicted by red circles, while blue squares indicate unpaired elements used for boundary determination

Consequently, sparsification improves the computation of $W^p$, $W\!M^p$ and $W\!M^2$. The corresponding sparsified version are

$$\begin{aligned} {\widehat{W}}^\text {p}(i,j)&= \min \{\, W(i,j-1); \min _{{[k,j]}\text { is candidate},\, k>i} W(i,k-1) + V(k,j) \,\}  \\ \widehat{W\!M}^\text {p}(i,j)&= \min \{\, W\!M(i,j-1) + c; \min _{{[k,j]}\text { is candidate},\, k>i }c\cdot (k-i) + V(k,j) \,; \widehat{W\!M}^{2}(i,j)\} \\ \widehat{W\!M}^{2}(i,j)&= \min \{\, \widehat{W\!M}^{2}(i,j-1) + c; \min _{{[k,j]}\text { is candidate},\, k>i} W\!M(i,k-1) + V(k,j) \,\}, \end{aligned}$$

where candidates ${[k,j]}$ correspond to the not optimally decomposable subsequences $S_{k,j}$ (in either situation: general structures or structures inside of multi-loops), i.e. ${[i,j]}$ is a *candidate* iff $V(i,j) < {\widehat{W}}^\text {p}(i,j)$ or $V(i,j)+b < \widehat{W\!M}^\text {p}(i,j)$. Similarly, the modified versions of the recursions can be found in Fig. 3.

### Time and space complexity of sparsified energy minimization

Will and Jabbari showed that following the above algorithm, $W(1,n)$ can be calculated in $O(n^2+nZ)$ time, where $Z$ is the total number of *candidates*. While the MFE structure in the Zuker and Stiegler algorithm can be trivially reconstructed following a traceback procedure, this is not the case if sparsification is used for improving time *and* space as in the SparseMFEFold algorithm (and our novel algorithms). To improve the space complexity, sparsification avoids storing all entries of the energy matrix. The idea is to store the candidates and as few additional matrix entries as possible. A specific challenge is posed by the decomposition of interior loops (the single most significant major complication over base pair maximization, see [13]). For this reason, Will and Jabbari introduced *trace arrows* for cases, where the trace cannot be recomputed efficiently during the traceback procedure; they discussed several space optimization techniques, such as avoiding trace arrows by rewriting the MFE recursions, and removing trace arrows as soon as they become obsolete. Due to such techniques, SparseMFEFold requires only linear space in addition to the space for candidates and trace arrows; its space complexity is best described as $O(n+T+Z)$, where *T* is the maximum number of trace arrows.

## Dangles

Recall that sparsification was discussed before (e.g., in SparseMFEFold) only for the simplest and least accurate variant of the Turner model, namely the one without dangling end contributions. Before we improve this situation, let’s look in more detail at dangling ends and different common ways to handle them. Specifically, we discuss different *dangle models* “no dangle” (model 0), “exclusive dangle” (model 1), and “always dangle” (model 2) as implemented by RNAFold of the Vienna RNA package (and available via respective command line options -d0, -d1, and -d2).

Dangling end contributions occur only at the ends of stems (either in multiloops or externally) due to stacking interaction between the closing base pair of the stem and one or both immediately adjacent unpaired bases. In contrast, dangling end terms are not considered within (interior loops of) stems by the energy model.

We present modified DP recursions in order to reflect precisely where and how dangling ends are taken into account. Therefore, in preparation, let’s replace $V$ in the Equations (1) and (4) of the free energy minimization recursions of Section “Preliminaries” by a new function $V^{\text {d}}$. The dangle models differ in the exact definition of $V^{\text {d}}$.

1' $$\begin{aligned} W(i,j)&= \min \{\, W^p(i,j), V^{\text {d}}(i,j) \,\} \end{aligned}.$$

4' $$\begin{aligned} W\!M(i,j)&= \min \{\, W\!M^p(i,j), V^{\text {d}}(i,j) + b \,\} \end{aligned}.$$

Note that in the energy model, dangling ends can also occur at the inner ends of helices that close a multi-loop. These dangles can be handled directly in the recurrence of $V(i,j)$; specifically, in the subcase where *i*.*j* closes a multi-loop.

### No dangles

In the simplest model “no dangle”, dangling ends are ignored. We achieve this by defining

no dangle $$\begin{aligned} V^{\text {d}}(i,j) := V(i,j) \end{aligned}.$$

While easy to implement, it is clearly wrong to ignore dangling end contributions, and this has a significant negative effect on the prediction accuracy compared to the other dangle models [34–36].

### Always dangle

A second relatively simple way is to apply a 53’ dangle energy at both ends of a stem (both 5’ and 3’ ends), assuming that stem ends always dangle with their adjacent bases. As a strong simplification, in this model, one disregards whether the bases are paired and/or dangle with a different stem (either case would actually make them unavailable for dangling).

This dangle model allows the dangling ends to have a thermodynamic influence while keeping the model easy to implement as neither the conflicting adjacent nucleotides nor the energies of single dangle have to be tracked; it only requires knowledge of the bases on the 3’ and 5’ sides of a base pair. Formally, we implement $V^{\text {d}}$ as

always dangle $$\begin{aligned} V^{\text {d}}(i,j) := V(i,j)+ {\text {dangle}}_{53}(i,j) \end{aligned}.$$

Moreover, we add the appropriate dangle contribution when closing a multi-loop in Eq. (3) in the last case of the $V$-recurrence of Eq. (3). The term $W\!M^2(i+1,j-1)+a$ is rewritten to

always dangle, ML closing $$\begin{aligned} W\!M^2(i+1,j-1)+a +{\text {dangle}}_{53}(i+1,j-1) \end{aligned}.$$

### Exclusive dangling

The most complex but general secondary structure dangle model, “exclusive dangle” considers both single and double unpaired nucleotides adjacent to a stem. Furthermore, the model does not allow shared dangling ends i.e. no base can be used simultaneously in two dangles (in other words, adjacent unpaired bases dangle *exclusively* with a single stem end). As the restriction requires tracking of unpaired bases, *V*(*i*, *j*) places the possible unpaired bases at *i* and *j* and looks at the adjacent *V* energies. As this requires knowledge of energies adjacent to the current bases being looked at, this inherently causes difficulty in sparsification.

exclusive dangle $$\begin{aligned} V^{\text {d}}(i,j) := \min {\left\{ \begin{array}{ll} V(i,j)\\ V(i+1,j)+ {\text {dangle}}_{5}(i) \\ V(i,j-1)+ {\text {dangle}}_{3}(j)\\ V(i+1,j-1) + {\text {dangle}}_{53}(i,j) \end{array}\right. } \end{aligned}$$

Moreover, we consider dangles at the closing of a multi-loop. In this model, the case $W\!M^2(i+1,j-1)+a$ in the minimization of Eq. (3) is replaced by (the minimum of) four different cases:

exclusive dangle, ML closing $$\begin{aligned} \min {\left\{ \begin{array}{ll} W\!M^2(i+1,j-1)+a\\ W\!M^2(i+2,j-1)+a + {\text {dangle}}_{3}(i) \\ W\!M^2(i+1,j-2)+a + {\text {dangle}}_{5}(j) \\ W\!M^2(i+2,j-2)+a + {\text {dangle}}_{53}(i,j) \\ \end{array}\right. } \end{aligned}$$

## Space-efficient sparsification with exclusive dangles is non-trivial

We approach our main motivation for this work, which is to study and solve the issues of sparsification in the exclusive dangle model (dangle model 1). Let’s thus start by applying the idea of sparsification (Section “Preliminaries”) straightforwardly to the Recursion (2) (where $W$ and $V^{\text {d}}$ are defined for exclusive dangles).

We quickly come up with the equation:

$$\begin{aligned} {\widehat{W}}^\text {p}(i,j)&= \min \{\, W(i,j-1); \min _{{[k,j]}\text { is ed-candidate}, k>i} W(i,k-1) + V^{\text {d}}(k,j) \,\}, \end{aligned}$$

but we would still have to define *ed-candidate* (exclusive dangle candidate) to make this work. We could define: [i,j] is an *ed-candidate* iff $V^{\text {d}}(i,j)<{\widehat{W}}^\text {p}(i,j)$, where the correctness of sparsification holds to a sparsification-typical triangle inequality argument (Section “Preliminaries”).

Expanding $V^{\text {d}}$ shows that this is not the only possible path to sparsifying the recursion. We could consider

$$\begin{aligned} {\widehat{W}}^\text {p}(i,j)&= \min {\left\{ \begin{array}{ll} W(i,j-1)\\ \min _{{[k,j]}\text { is ed0-candidate}, k>i} W(i,k-1) + V(i,j)\\ \min _{{[k,j]}\text { is ed5-candidate}, k>i} W(i,k-1) + V(i+1,j)+ {\text {dangle}}_{5}(i) \\ \min _{{[k,j]}\text { is ed3-candidate}, k>i} W(i,k-1) + V(i,j-1)+ {\text {dangle}}_{3}(j)\\ \min _{{[k,j]}\text { is ed53-candidate}, k>i} W(i,k-1) + V(i+1,j-1) + {\text {dangle}}_{53}(i,j) \end{array}\right. } \end{aligned}$$

with different sets of candidates for all four cases. However, storing all these candidate sets (recall that there is even a second recursion that needs to be sparsified) is easily prone to compromising any space benefits due to sparsification in practice.

The transfer of the techniques from [15] brings even more problems, since due to such definitions, candidates [i,j] do not necessarily correspond to subsequences that have closed optimal structures. Will and Jabbari strongly exploited this fact for their strong space savings.

Even considering our definition of an ed-candidate, we still run into the challenge of to tracing back to the corresponding base pair. With just the dangle energy, this poses issues as an ed-candidate can be one of four cases.

### Lemma 1

*In the exclusive dangle model, storing only the energy of each ed-candidate is not sufficient to correctly trace back from the candidate*.

### Proof

Concretely, for the loop-based Turner 2004 energy model [37] with exclusive dangles, consider the following RNA sequence *S* of length 12 with its MFE structure:
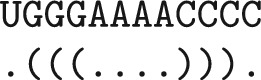


In the calculation of $W(1,12)$, the recurrences unfold to $W(1,12) =W(1,1) + V^{\text {d}}(2,12) = W(1,1) + V(2,11) + {\text {dangle}}_{3}(12) = \dots =-2.9$ kcal/mol, i.e. it is optimal to assume dangling of base pair (2, 11) to the right.

In a non time- and space-sparsified algorithm, recomputing $V^{\text {d}}$ from $V$ adjacent energies would be trivial. However, due to space sparsification, the values of $V$ are generally unavailable in the trace-back phase. In the constructed example, recomputation would require us to know $V(2,12)$, $V(2,11)$, $V(3,12)$, and $V(3,11)$. Thus, under the assumption of the lemma, the optimal dangling cannot be efficiently recomputed for a candidate like [2,12]. $\square$

In our preceding work, SparseMFEFold [15], trace arrows were introduced to trace back to non-candidate values necessary to the structure within the interior loop case: $V^{\text {il-cand}}(i,j)$. Trace arrows that point to candidates are not stored as they can be avoided by minimizing over candidates as seen in Eq. (7).

7 $$\begin{aligned} V^{\text {il-cand}}(i,j) = \min _{\begin{array}{c} i<p<q<j\\ p-i+j-q-2\le M\\ {} {[{p,\,q}]\text {is candidate}} \end{array}} \,\mathcal {I}(i,j;p,q) + V(p,q). \end{aligned}$$

Consequently, finding the inner base pair of a loop through a candidate relies on the energy saved being *V*(*p*, *q*). However, as shown in Eq. (exclusive dangle, ML closing), the dangle energy could be *V*(*p*, *q*), $V(p+1,q)$, $V(p,q-1)$, or $V(p+1,q-1)$. Replacing the stored energy within a candidate with $V^{\text {d}}$ may conflict with the interior loop calculation. Recomputation of the $V$ values required for $V_{d}$ would negate the sparsification benefit. In summary, there is no easy or direct way to save the $V$ energy required for the interior loop as well as the $V^{\text {d}}$ energy required for a multi-loop or external loop within the current candidate structure.

### Lemma 2

*The minimization over inner base pairs in the recursion of* $V$ *cannot be restricted to candidates in the same way as in SparseMFEFold*.

### Proof

Again consider the loop-based Turner 2004 energy model. There is a sequence *S* and $1\le i<j\le n$, such that $V^{\text {d}}(p,q) <$ $V(p,q)$, but there is no way to trace back to *p* and *q* from *i* and *j*, namely, consider the RNA sequence *S* of length 19 with its MFE structure:
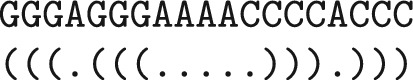


The optimal recursion case of $V(3,17)$ forms the interior loop closed by 3.17 with inner base pair 5.15, because $V(5,15) = -2.4$ kcal/mol and V(3,17) = $\mathcal {I}(3,17;5,15) + V(5,15) = -1.5$ kcal/mol.

The space optimization of SparseMFEFold removes trace arrows to candidates since the trace-back to candidates can be reconstructed based on candidate energies (compare Eq. (7)).

In the way of SparseMFEFold, we would not store a trace arrow pointing to 5.15 from ${[3,17]}$, since ${[5,15]}$ is a candidate. However, without a trace arrow, we would not reconstruct the correct trace. This happens, since the optimal structure in the subsequence 5..15, GGGAAAACCCC, would be (((....))). due to the 3’ dangle ($V^{\text {d}}(5,15) = -2.9$ kcal/mol). Consequently, tracing back the optimal path from $V^{\text {d}}(5,15)$ wrongly introduces a base pair at 5.14. $\square$

## SparseRNAFolD

SparseRNAFolD combines the power of sparsification and a general energy model including dangle energies to achieve a fast and highly accurate RNA pseudoknot-free secondary structure prediction. To this end, we started with the sparsified dynamic programming recurrences of SparseMFEFold (which implements the “no dangles” model), rewriting and revising them to accommodate various dangle energies.

### “Always dangle” model

Recall that “always dangle” model considers both the 5’ and 3’ ends of a branch of a multi-loop or external loop for dangle contributions. The addition of this model is trivial, with no change necessary to the recurrences of the SparseMFEFold. Note that, as mentioned earlier, this model ignores overlapping cases and may overcount the contributions of dangles.

### “Exclusive dangle” model

As mentioned in Section Space-efficient sparsification with exclusive dangles is non313 trivial, accounting for the “exclusive dangle” model is non-trivial when dealing with candidates, as ed-candidates do not hold enough information to identify the direction of dangles. To alleviate this problem, we provide three different strategies, as described below. Each strategy has its pros and cons and should be selected based on the application.

In order to handle the changes for exclusive dangles, we extend the candidate data structure. A candidate base pair, ${[i,j]}$ as implemented in SparseMFEFold, holds *i*, the start position, and the energy $V(i,j)$ as a tuple $(i,V(i,j))$ and is stored at the *j*th index of the candidate list. Our extensions to candidate structures involves including the energy values for $W$ and $W\!M$ in the candidate tuples as $(i,V(i,j),W(i,j),W\!M(i,j))$. The modification reflects the need to store more information about the dangles positions and directions.

#### Strategy 1: trace arrow implementation

As the first strategy to trace an ed-candidate to its position, we used modified trace arrows. We refer to this strategy as **SparseRNAFolD-Trace**.

Recall that in SparseMFEFold, a trace arrow structures were introduced to identify energy matrix entries that are necessary for calculating the energy of internal loops but are not kept as candidates. Here, we define *ed-trace-arrows* to hold information about dangle positions to aid with the traceback procedure from ed-candidates. In particular, in the sparse fold reconstruction procedure of SparseRNAFolD, an ed-trace-arrow is checked for a chosen ed-candidate within $W$, $W\!M$, and $\widehat{W\!M}^{2}$ to adjust the energy and position of the base pair as required. The drawback of this strategy comes from the innate inefficiencies of the trace arrows, meaning an increase in space usage. Recall that within SparseMFEFold, we used strategies such as garbage collection and trace arrow avoidance to save space. These strategies are not, however, possible for SparseRNAFolD-Trace, as an ed-candidate cannot be excluded from the optimal MFE path, and an ed-trace-arrow is therefore required for every ed-candidate.

##### Bit encoding

Within the second and third strategies, as explained next, we employed bit encoding and bit decoding to store the information about the dangle within the energy values to reduce space usage. Currently, energy values are stored as 32-bits *int* data type. We note that the maximum expected bit usage for the energy value of an RNA sequence of up to 20000 bases is about 13 bits. We employed a bit shift to store the dangle type in the first two bits of the *V* entries, referred to as $V_{enc}$, and represented in Eq. (8).

8 $$\begin{aligned} V_{enc} = (V\ll 2)\ \mathbin {|} \ dangle \end{aligned}$$

Bit decoding technique was used to retrieve the energy value and type/direction of dangle contributions. Bit decoding was done in two steps. Shifting the encoded energy, $V_{enc}$, two bits forward gave back the energy, *V* (see Eq. 9).

9 $$\begin{aligned} V= V_{enc} \gg 2 \end{aligned}$$

The dangle type is found in the first two bits; no dangle is represented with a “00” in bits; a 5’ dangle with a “01”; a 3’ dangle with a “10”; and a 53’ dangle with a “11”. The dangle type is decoded using a bit-wise AND with “11” to only keep the first two bits of the encoded energy, as represented in Eq. (10).

10 $$\begin{aligned} dangle = V_{enc}\ \mathbin { \& \& }\ 11 \end{aligned}$$

#### Strategy 2: Bit encoding with candidate extension

As the second strategy, we used bit encoding within the $W$ and $W\!M$ entries of the ed-candidate data structure. We refer to this strategy as SparseRNAFolD-Standard. This implementation of bit encoding was utilized in $W$ and $W\!M$ entries, as other loop types do not deal with dangles.

#### Strategy 3: Bit encoding with altered candidate

As the third strategy, we further optimize for space by reducing the candidate size. To reduce candidate size, we stored energy values in ed-candidates in $W$ and $W\!M$ as $V^{\text {d}}$ minus the dangle energy. We refer to this strategy as **SparseRNAFolD-Triplet**. This strategy allows for the correct identification of dangle types regardless of energy parameters used. Note that currently, in the Turner 2004 energy model, the parameter values for 53’ dangle for an external loop and multi-loop are the same. These values may be further estimated and revised in future energy models. The extra calculations to retrieve the $V^{\text {d}}$ value ensure the accuracy of the result in the event of such a change.

As we only require constant space for each candidate, the asymptotic time and space complexity can be expressed analogously to SparseMFEFold [15] with the time as $O(n^2 + nZ)$ and $O(n+Z+T)$ with $Z<<n^2$ – where *Z* is the number of candidates in SparseRNAFolD and *T* is the number of trace arrows in SparseRNAFolD.

### Compared methods

To evaluate the performance of our SparseRNAFolD, we compared it to two of the best-performing methods for prediction of pseudoknot-free RNA secondary structure, namely RNAFold [18] and LinearFold [19].

### RNAFold

RNAFold is part of the Vienna RNA package [18]. As discussed in Section “Dangles”, RNAFold is an $O(n^3)$ time and $O(n^2)$ space pseudoknot-free RNA secondary structure prediction algorithm. It takes an RNA sequence as input and provides the MFE structure as output. RNAFold is well-maintained and highly optimized and is used here as a benchmark for a fast implementation of the Zuker and Steigler-type MFE algorithm.

### LinearFold

LinearFold [19] is a pseudoknot-free RNA secondary structure prediction algorithm that uses heuristic techniques to run in linear time and space. As the main goal of sparsification is to speed up the time and space complexity of MFE prediction, we set out to investigate how our SparseRNAFolD compares in practice to LinearFold with better asymptotic complexities.

LinearFold employs two techniques to reduce its time and space complexity to *O*(*n*), namely *beam pruning* and *k-best parsing*. Both methods aim to prune the structure path to optimal cases only. Beam pruning works by only keeping a predetermined number (specified by the beam width, *b*) of the optimal states. Within LinearFold, best sets are kept for each possible loop type as defined in the Zuker algorithm: hairpin, multi-loop fragments, and internal loop. Through beam pruning, time complexity is reduced to $O(nb^2)$ and the space to *O*(*nb*) where *b* is the beam width. K-best parsing further reduces the time to $O(nb \log (b))$. We note that due to the heuristic nature of the LinearFold algorithm, it does not guarantee finding the MFE structure for a given RNA sequence.

## Experimental design

We implemented SparseRNAFolD in C++. All experiments were performed using an Azure virtual machine. The virtual machine contained 8 vCPUs with 128 GiB of memory.

### Dataset

We used the original dataset from SparseMFEFold [15]. This dataset is comprised of 3704 sequences in 6 different families selected from the RNAstrand V2.0 database [38]. The smallest sequence is 8 nucleotides long, while the largest is 4381 nucleotides long.

### Energy model

We used the energy parameters of the Turner 2004 energy model [37, 39], as implemented in the ViennaRNA package [18].

### Accuracy measures

The number of *true positives* (TP) is defined as the number of correctly predicted base pairings within the structure. The number of *false positives* (FP), similarly, is the number of predicted base pairs that do not exist in the reference structure. Any base missed in the prediction that corresponds to a pairing in the reference structure is a *false negative* (FN).

We evaluate the performance of algorithms based on three measures: sensitivity, positive predictive value (PPV), and their harmonic mean (F-measure).

11 $$\begin{aligned}{} & {} Sensitivity = \frac{TP}{TP + FN} \end{aligned},$$

12 $$\begin{aligned}{} & {} PPV = \frac{TP}{TP + FP} \end{aligned},$$

13 $$\begin{aligned}{} & {} F_{measure}= \frac{2\cdot PPV \cdot Sensitivity}{PPV+Sensitivity} \end{aligned}.$$

### Proof of concept with RNAFold

As a proof of concept for the correct implementation of dangle energy models (i.e., “always dangle” and “exclusive dangle”), we assessed SparseRNAFolD against RNAFold. As the MFE structure may not be unique, we restricted our assessment to the MFE value obtained by each method. We found that the MFE predicted by SparseRNAFolD and RNAFold was the same. Details of the results can be found in our repository.

## Results

We measured runtime using user time and memory using the maximum resident set size.

### Alternative models

**Fig. 4:**
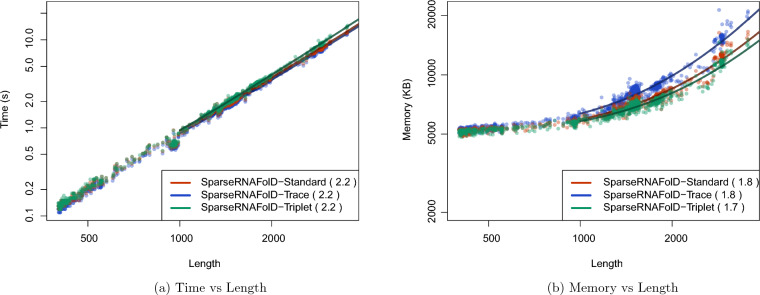
We plot the results of the three versions of SparseRNAFolD when given RNA sequence only as input against each other and an “exclusive dangle” model based on the dataset. **a** Memory Usage (maximum resident set size in KB) versus length (log-log plot) over all benchmark instances. The solid line shows an asymptotic fit $(c_1+c_2n^x)$ for sequence length *n*, constants $c_1$, $c_2$, and exponent *x* for the fit. We ignored all values $< 1000$. **b** Run-time (s) versus length (log-log plot) over all benchmark instances. For each tool in both plots, we report (in parenthesis) the exponent *x* that we estimated from the benchmark results; it describes the observed complexity as $\Theta (n^x)$

We start by comparing the three different implementations of SparseRNAFolD. SparseRNAFolD-Standard was found to be in the middle in terms of memory and time. The effect of additional trace arrows in SparseRNAFolD-Trace had a $27\%$ increase in memory usage on the largest sequence compared to SparseRNAFolD-Standard. However, the increase in computation from the bit encoding only resulted in a $5\%$ increase in time on the largest sequence. We find a similar effect when comparing SparseRNAFolD-Standard and SparseRNAFolD-Triplet. The altered triplet structure reduced the memory by $9\%$ but increased the time by $10\%$ due to extra computation. These are highlighted in Fig. 4.

### Comparison with LinearFold and RNAFold

**Fig. 5:**
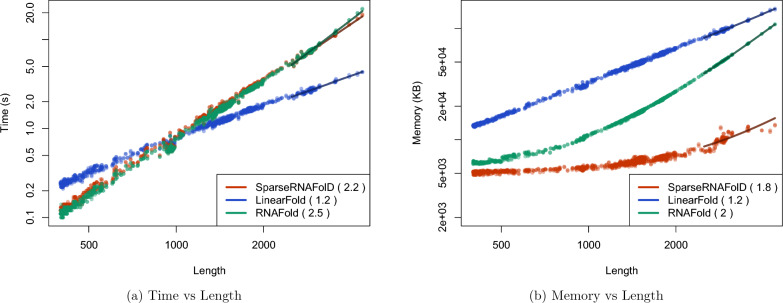
We plot the results of SparseRNAFolD-Standard against two state of the art algorithms: RNAFold and LinearFold when given RNA sequence only as input against each other and an “always dangle” model on our dataset and the dinucleotide shuffled version of our dataset. **a** Memory Usage (maximum resident set size in KB) versus length (log-log plot) over all benchmark instances. The solid line shows an asymptotic fit $(c_1+c_2n^x)$ for sequence length n, constants $c_1$, $c_2$, and exponent *x* for the fit. We ignored all values $< 1000$. **b** Run-time (s) versus length (log-log plot) over all benchmark instances. For each tool in both plots, we report (in parenthesis) the exponent *x* that we estimated from the benchmark results; it describes the observed complexity as $\Theta (n^x)$

When comparing SparseRNAFolD-Standard with LinearFold and RNAFold, we look at the “always dangle” model, as LinearFold does not implement the “exclusive dangle” model.

We first compared the three algorithms by their predictive accuracy (F-measure). For comparison, we selected all sequences from our dataset whose structure was available on RNAstrand. We further constrained it to sequences that contained hairpins greater than 3 and no pseudoknots. This resulted in 986 sequences. We found that SparseRNAFolD-Standard had a marginally better, but not significant, average F-measure of 0.6394 compared to 0.6391 of LinearFold. As described in Section “Proof 450 of concept with RNAFold”, RNAFold and SparseRNAFold-Standard are identical in predictive accuracy.

We then assessed their time and space usage. To increase the size of our dataset for this testing, we included a dinucleotide shifted version of our dataset in our test data. We then constrained the size of sequences to those $>400$. The maximum time and memory used by LinearFold on this dataset were 3.34 s and 118, 848 KB. The maximum time and memory used by RNAFold were 22.26 s and 109136 KB. In contrast, the maximum time and memory spent by SparseRNAFold were 18.32 s and 13,000 KB, respectively. This is illustrated in Fig. 5a, b. The results show that SparseRNAFolD-Standard uses far less memory on even the largest pseudoknot-free sequences in our dataset. Note that the maximum resident set size is nine times lower than that of LinearFold and eight times lower than that of RNAFold. RNAFold’s time remained consistent with SparseRNAFolD-Standard until longer sequences where it falls behind. LinearFold, whose time complexity is $O(nb\log (b))$, where n is the length of the sequence and b is the beam width, did perform faster than SparseRNAFolD-Standard as the length of the sequence increased. However, we did find that SparseRNAFolD-Standard outperformed LinearFold in practice for sequences of up to about 1000 nucleotides.

### Highlighting RNAFold

To highlight the difference in space between RNAFold and SparseRNAFolD-Standard, we selected 81 sequences from our dataset with size greater than or equal to 2500. The sequence with the maximum length in the set was 4381 nucleotides long.

**Table 1:** We tabulate the results of the comparison between RNAFold and SparseRNAFolD-Standard when given only sequences with length $>2500$ from our dataset as input and using the “exclusive dangle” model

	Run-time (s)		Memory: resident set size (KB)	
	RNAFold	SparseRNAFolD	RNAFold	SparseRNAFolD
Minimum	5.04	5.36	40,148	8832
Median	7.28	7.86	51,284	12,592
Maximum	22.08	18.32	109,040	16,836

We looked at time (s) and memory (maximum resident set size in KB) for the minimum, median and maximum length sequence within the constrained dataset

As seen in Table 1, while SparseRNAFolD-Standard’s runtime is comparable to RNAFold’s, its memory consumption is about five times lower.

### Candidate comparison

**Fig. 6:**
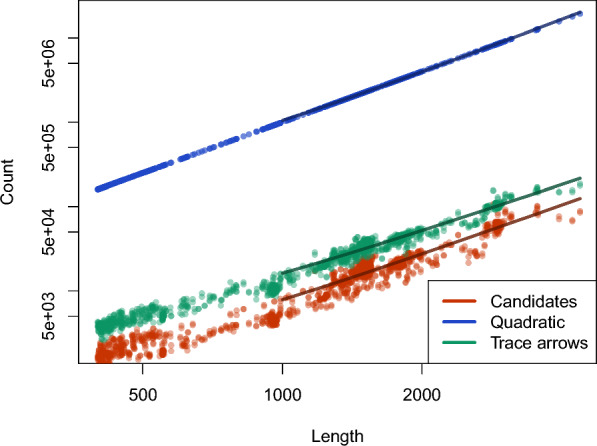
We plot the results of the number of candidates and trace arrows compared to quadratic space. ‘Quadratic’ shows the count within an $n \times n$ matrix as it would be given quadratic space. In contrast, ‘Candidates’ and ‘Trace arrows’ show the contrasting number for the same length

In order to illustrate the effectiveness of candidates in terms of memory consumption, we plotted the relationship between the number of candidates and trace arrows, against the quadratic space, using the dataset that includes dinucleotide shifted elements. To emphasize the upper limit of candidate usage when executing SparseRNAFolD-Standard, we employ the “exclusive dangle” model.

For a more meaningful comparison, we juxtapose the counts of candidates and trace arrows with the count obtained from a single quadratic matrix. It is important to note that the majority of algorithms employing quadratic space make use of multiple quadratic matrices. Considering this aspect, we discovered that, on average, the disparity in count between the number of candidates and trace arrows with quadratic space was approximately a factor of 100. Figure 6 highlights that the increase in candidates is consistent with the increase in length.

### Folding with hard constraints

As partial information on structures has become more available and is extensively used for better prediction of possibly pseudoknotted structures [40, 41], we further extend our evaluation of the SparseRNAFolD versions to cases where we are folding with hard constraints [23] in addition to the RNA sequence.

**Fig. 7:**
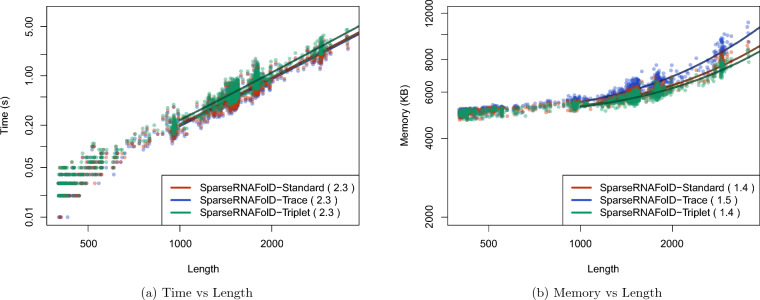
We plot the results of the three versions of SparseRNAFolD when given an RNA sequence, an “exclusive dangle” model, and a random pseudoknot-free structure as input against each other based on our dataset. **a** Memory usage (maximum resident set size in KB) versus length (log-log plot) over all benchmark instances. The solid line shows an asymptotic fit $(c_1+c_2n^x)$ for sequence length *n*, constants $c_1$,$c_2$, and exponent *x* for the fit. We ignored all values $< 1000$. **b** Run-time (s) versus length (log-log plot) over all benchmark instances. For each tool in both plots, we report (in parenthesis) the exponent *x* that we estimated from the benchmark results; it describes the observed complexity as $\Theta (n^x)$

To study the effect of hard structure constraints on the efficiency of our sparsified folding algorithm, for each sequence, a pseudoknot-free constraint structure was generated. The structure was generated by taking two random indices at a time from the sequence. If the two bases could pair, were at least 3 bases apart, and did not form a pseudoknot with the other base pairs, the base pair was added to the constraint structure. In order to avoid overpopulating the constraint structure, the number of base pairs in a constraint structure was capped at $0.5\times \log _2(length)$. This resulted in an average of 3–7 base pairs per sequence. There was a noticeable decrease in time and space when a constraint structure was provided in addition to an RNA sequence. Between RNA sequence only as input and sequence as well as a constraint structure, SparseRNAFolD saw a $67\%$ decrease in time and a $40\%$ decrease in memory. As the constraint structure reduced the number of candidates for a sequence, the difference in memory was less apparent between the models. SparseRNAFolD-Standard had a $6\%$ increase in time from SparseRNAFolD-Trace but a $15\%$ decrease in memory on the largest sequence. From SparseRNAFolD-Standard to SparseRNAFolD-Triplet, there was an $8\%$ decrease in memory but a $13\%$ increase in time. Note that even when reducing the number of candidates, the increase in time from Standard to Triplet was greater by $3\%$. This can be seen in Fig. 7.

### Modification of internal loop logic

In MFE-based dynamic programming algorithms, the calculation of the internal loop stands out as the primary time-consuming element in the prediction process. This holds particularly true in the context of sparsified prediction, as the internal loop remains the singular aspect where sparsification struggles to enhance runtime during energy calculations. Given this bottleneck, any enhancement to the internal loop logic signifies a substantial improvement in sparsified algorithms.

To accommodate constraint folding, a logic check was incorporated to verify that the interior base pair does not violate the specified structural constraint. Given the higher frequency of occurrences of internal loops compared to non-internal loop calculations, the impact of frequent branch mispredictions on processing time is substantial. Specifically, a typical branch instruction requires 0–2 clock cycles, whereas a branch misprediction can lead to a significant latency of 12–25 clock cycles, depending on the processor [42]. To mitigate the impact of branch misprediction, we opted to reconfigure the logic check, shifting its reliance from branching to operations involving addition and bit manipulations. We refer to this version of SparseRNAFold as SparseRNAFold V2.0. The initial format of the logic check involves nested *if statements* within the *for loops*. The first *if statement* verifies the absence of paired bases between the two opening bases of the base pairs, while the second *if statement* ensures the presence of only unpaired bases between the two closing bases—see Algorithm 1. Nevertheless, we can achieve equivalent functionality through bit manipulation, eliminating the need for explicit *if statements*.

**Algorithm 1:**
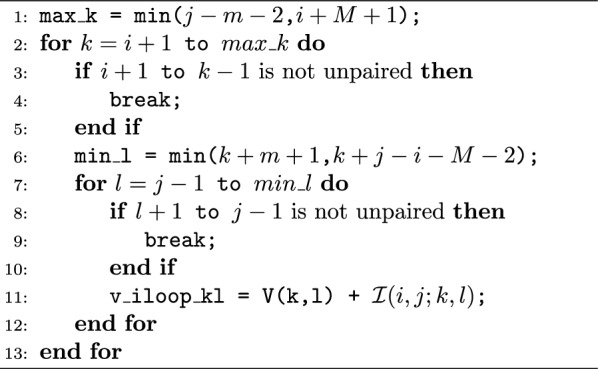
Original internal loop

1. Fundamentally, our objective is to attain the energy value when feasible and assign infinity otherwise. With this perspective, we can conceptualize this process as adding 0 when achievable and incorporating infinity when the energy value is not attainable.
2. The second key point to keep in mind is that, for a signed integer, the representation of 0 entails the signed bit as 0, followed by all subsequent bits as 0. Conversely, the representation of $-1$ involves the signed bit as 1, with all subsequent bits set to 1.

**Algorithm 2:**
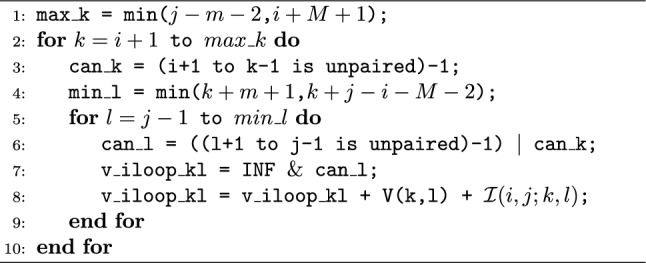
Modified internal loop

In our initial *if statement*, we store the boolean indicating the absence of bases on the left as an integer and subtract 1, guaranteeing a result of either 0 or $-1$. The same process is then applied to the right side, and the results are combined using a bitwise OR operation. If both sides permit pairing, the result is 0; otherwise, it is $-1$. This result is subsequently bitwise ANDed with a predefined large value representing infinity. Consequently, if the result was 0, the outcome is 0, and if the result was $-1$, it is set to infinity. Finally, we add our energy value to this outcome. This sequence of operations ensures that both sides permit pairing, all without the need for explicit branching via an if statement—see Algorithm 2.

We observed an approximately $17\%$ reduction in prediction time for the largest sequence in the dataset (10241) when comparing the original SparseRNAFolD to SparseRNAFold V2.0.

**Fig. 8:**
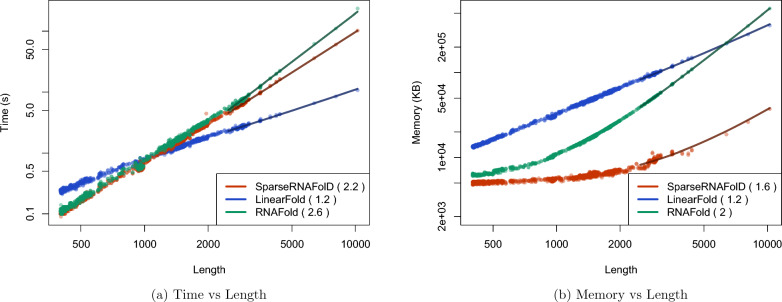
We plot the results of SparseRNAFolD V2.0 against two state of the art algorithms: RNAFold and LinearFold when given RNA sequence only as input against each other and an “always dangle” model on our dataset and the dinucleotide shuffled version of our dataset and three added sequences of length: 6380, 8082, and 10241. **a** Memory Usage (maximum resident set size in KB) versus length (log-log plot) over all benchmark instances. The solid line shows an asymptotic fit $(c_1+c_2n^x)$ for sequence length *n*, constants $c_1$,$c_2$, and exponent *x* for the fit. We ignored all values $< 1000$. **b** Run-time (s) versus length (log-log plot) over all benchmark instances. For each tool in both plots, we report (in parenthesis) the exponent *x* that we estimated from the benchmark results; it describes the observed complexity as $\Theta (n^x)$

Since the internal loop represents an area within sparsified algorithms that cannot be sparsified, this improvement can be applied not only to SparseRNAFold V2.0 but also to other MFE-based dynamic programming algorithms (Fig. 8).

### Performance on very large sequences

The advantage of utilizing heuristic approaches such as LinearFold lies in their capability to predict larger sequences efficiently, attributed to their low time complexity. However, this advantage comes with the trade-off that they cannot guarantee the prediction of the MFE structure for the given sequence. To underscore the effectiveness of sparsification and the enhancements in internal loop logic, we conducted an analysis on the SARS-CoV-2 RNA, which spans a length of 29, 903 bases. As seen in Table 2, although SparseRNAFolD V2.0 exhibited a longer prediction time for predicting the MFE structure of this RNA, it delivered a structure with lower free energy while utilizing only $25.5\%$ of the memory at this extended length.

**Table 2:** We tabulate the results of the comparison between LinearFold and SparseRNAFolD V2.0 when given the SARS-COV-2 sequence of length 29,903 as input and using the “always dangle” model

	SparseRNAFolD V2.0	LinearFold
Energy (kcal/mol)	− 8787.50	− 8476.20
Run-time (s)	1127.92	27.67
Memory: resident set size (KB)	274,704	1,076,904

We looked at the energy (kcal/mol), time (s) and memory (maximum resident set size in KB)

## Conclusions

In this work, we introduced SparseRNAFolD, a sparsified MFE RNA secondary prediction algorithm that incorporates dangles contribution to the energy calculation of a sparsified method. We showed that while “no dangle” and “always dangle” models were easy to incorporate into the existing algorithms, “exclusive dangle” introduces non-trivial challenges that need calculated changes to the sparsified recursions to alleviate. We identified three strategies to implement dangle contributions: SparseRNAFolD-Trace which utilizes additional trace arrows; SparseRNAFolD-Standard, which incorporates bit encoding as well as extension to the definition of candidate structures; and SparseRNAFolD-Triplet, which, similar to the SparseRNAFolD-Standard, utilizes bit encoding but modifies candidate energy calculation in anticipation of possible change in parameters in the future. Comparing these three versions on a large dataset, we concluded that the SparseRNAFolD-Triplet implementation is the most efficient in terms of memory, and SparseRNAFolD-Trace is the most efficient in terms of time. These two versions showcase how space and time trade-offs can improve performance for a specific application. The SparseRNAFolD-Standard version provides a middle ground for improvement in both time and space and has been chosen as the standard implementation of our algorithm. While guaranteeing the MFE structure and matching the energy of RNAFold, our SparseRNAFolD is on par with LinearFold on memory usage and run time for sequences up to about 1000 bases. This provides a promising starting point to bring dangles contributions to pseudoknotted MFE structure prediction methods in which memory usage is the prohibitive factor [17].

Our results showcase the substantial difference in the number of candidates when compared to quadratic space. This provides an illuminating perspective on the space improvement achieved through sparsification.

We further assessed the effect of hard structural constraints on the performance of SparseRNAFolD, presenting significant improvements both in terms of time and space. We believe the significant improvement in time and space due to the limitation of search space by hard structural constraints can have a more pronounced impact on sparsified pseudoknotted MFE prediction, which is our ultimate goal.

We enhanced our initial algorithm by refining the internal loop logic, mitigating branch mispredictions through the elimination of conditionals and incorporating the same functionality via addition and bit manipulation. This optimization resulted in a notable $17\%$ improvement over the original code

Additionally, we demonstrated SparseRNAFolD V2.0’s proficiency in predicting extensive sequences, exemplified by its handling of the SARS-CoV-2 sequence comprising 29, 903 bases. Notably, our approach ensures the prediction of the MFE structure while consuming less memory compared to LinearFold.

Finally, memory consumption becomes a bottleneck for the prediction of MFE structure for long RNA sequences or MFE pseudoknotted structure prediction. Utilizing the power of computational servers, such restrictions have been somewhat alleviated. Sparsification provides improvements in both time and space requirements and can be used to bring computations back to personal computers, providing equal access to the existing technology. In addition, improvements in memory usage can improve use cases for computing clusters, as the amount of memory assigned to a computing node is also limited.

## Data Availability

The dataset supporting the conclusions of this article is available in the repository, https://github.com/mateog4712/SparseRNAFolD-RawData. SparseRNAFolD’s algorithm and detailed results are available at https://github.com/mateog4712/SparseRNAFolD.

## References

[1] Cruz JA, Westhof E. The dynamic landscapes of RNA architecture. Cell. 2009;136:604–9. 10.1016/j.cell.2009.02.003. 19239882 10.1016/j.cell.2009.02.003

[2] Kozak M. Regulation of translation via mRNA structure in prokaryotes and eukaryotes. Gene. 2005;361:13–37. 10.1016/j.gene.2005.06.037. 16213112 10.1016/j.gene.2005.06.037

[3] Mortimer SA, Kidwell MA, Doudna JA. Insights into RNA structure and function from genome-wide studies. Nat Rev Genet. 2014;15:469–79. 10.1038/nrg3681. 24821474 10.1038/nrg3681

[4] Warf MB, Berglund JA. Role of RNA structure in regulating pre-mRNA splicing. Trends Biochem Sci. 2010;35:169–78. 10.1016/j.tibs.2009.10.004. 19959365 10.1016/j.tibs.2009.10.004 PMC2834840

[5] Wilson TJ, Lilley DMJ. RNA catalysis—is that it? RNA. 2015;21:534–7. 10.1261/rna.049874.115. 25780127 10.1261/rna.049874.115 PMC4371269

[6] Holt CE, Bullock SL. Subcellular mRNA localization in animal cells and why it matters. Science. 2013;326:1212–6. 10.1126/science.1176488. 10.1126/science.1176488 PMC3785123 19965463

[7] Martin KC, Ephrussi A. mRNA localization: gene expression in the spatial dimension. Cell. 2009;136:719–30. 10.1016/j.cell.2009.01.044. 19239891 10.1016/j.cell.2009.01.044 PMC2819924

[8] Mathews DH, Turner DH. Prediction of RNA secondary structure by free energy minimization. Curr Opin Struct Biol. 2006;16(3):270–8. 10.1016/j.sbi.2006.05.010. 16713706 10.1016/j.sbi.2006.05.010

[9] Nowakowski J, Tinoco I. RNA structure and stability. Semin Virol. 1997;8(3):153–65. 10.1006/smvy.1997.0118. 10.1006/smvy.1997.0118

[10] Wexler Y, Zilberstein C, Ziv-Ukelson M. A study of accessible motifs and RNA folding complexity. J Comput Biol. 2007;14:856–72. 10.1089/cmb.2007.R020. 17691898 10.1089/cmb.2007.R020

[11] Salari R, Möhl M, Will S, Sahinalp SC, Backofen R. Time and space efficient RNA-RNA interaction prediction via sparse folding. In: Research in computational molecular biology. Berlin, Germany: Springer; 2010. p. 473–90. 10.1007/978-3-642-12683-3_31.

[12] Möhl M, Salari R, Will S, Backofen R, Sahinalp S. Sparsification of RNA structure prediction including pseudoknots. Algorithms Mol Biol. 5 (2010) 10.1186/1748-7188-5-39 10.1186/1748-7188-5-39 PMC3161351 21194463

[13] Backofen R, Tsur D, Zakov S, Ziv-Ukelson M. Sparse RNA folding: time and space efficient algorithms. J Discrete Algo. 2011;9:12–31. 10.1016/j.jda.2010.09.001. 10.1016/j.jda.2010.09.001

[14] Dimitrieva S, Bucher P. Practicality and time complexity of a sparsified RNA folding algorithm. J Bioinformat Comput Biol 10 (2012) 10.1142/S0219720012410077 10.1142/S0219720012410077 22809342

[15] Will S, Jabbari H. Sparse RNA folding revisited: space-efficient minimum free energy structure prediction. Algorithms for Molecular Biology **11** (2016) 10.1186/s13015-016-0071-y 10.1186/s13015-016-0071-y PMC4842305 27110275

[16] Jabbari H, Wark I, Mothentemagno C, Will S. Sparsification enables predicting kissing hairpin pseudoknot structures of long RNAs in practice. In: 17th International Workshop on Algorithms in Bioinformatics (WABI 2017). Leibniz International Proceedings in Informatics (LIPIcs), vol. 88, pp. 12–11213. Schloss Dagstuhl–Leibniz-Zentrum fuer Informatik, Oktavie-Allee, 66687 Wadern, Germany (2017). 10.4230/LIPIcs.WABI.2017.12

[17] Jabbari H, Wark I, Montemagno C, Will S. Knotty: efficient and accurate prediction of complex RNA pseudoknot structures. Bioinformatics. 2018;34:3849–56. 10.1093/bioinformatics/bty420. 29868872 10.1093/bioinformatics/bty420

[18] Lorenz R, Bernhart S.H, Siederdissen C, Tafer H, Flamm C, Stadler P.F, Hofacker I.L. ViennaRNA package 2.0. Algo Mol Biol. 2011;6. 10.1186/1748-7188-6-26 10.1186/1748-7188-6-26 PMC3319429 22115189

[19] Huang L, Zhang H, Deng D, Zhao K, Liu K, Hendrix DA, Mathews DH. Linearfold: linear-time approximate RNA folding by 5’-to-3’ dynamic programming and beam search. Bioinformatics. 2019;35:295–304. 10.1093/bioinformatics/btz375. 10.1093/bioinformatics/btz375 PMC6681470 31510672

[20] Hofacker IL, Stadler PF. Memory efficient folding algorithms for circular RNA secondary structures. Bioinformatics. 2006;22:1172–6. 10.1093/bioinformatics/btl023. 16452114 10.1093/bioinformatics/btl023

[21] McCaskill JS. The equilibrium partition function and base pair binding probabilities for RNA secondary structure. Biopolymers. 1990;29:1105–19. 10.1002/bip.360290621. 1695107 10.1002/bip.360290621

[22] Bompfünewerer AF, Backofen R, Bernhart SH, Hertel J, Hofacker IL, Stadler PF, Will S. Variations on RNA folding and alignment: lessons from Benasque. J Mathe Biol. 2008;56:129–44. 10.1007/s00285-007-0107-5. 10.1007/s00285-007-0107-5 17611759

[23] Lorenz R, Hofacker IL, Stadler PF. RNA folding with hard and soft constraints. Algo Mol Biol. 2016;11 (2016) 10.1186/s13015-016-0070-z 10.1186/s13015-016-0070-z PMC4842303 27110276

[24] Zuker M, Stiegler P. Optimal computer folding of large RNA sequences using thermodynamic and auxiliary information. Nucleic Acids Res. 1981;9:133–48. 10.1093/nar/9.1.133. 6163133 10.1093/nar/9.1.133 PMC326673

[25] Hofacker IL, Fontana W, Stadler PF, Bonhoeffer LS, Tacker M, Schuster P. Fast folding and comparison of RNA secondary structures. Chem Monthly. 1994;125:167–88. 10.1007/BF00818163. 10.1007/BF00818163

[26] Reuter J.S, Matthews D.H. RNAstructure: software for RNA secondary structure prediction and analysis. In: Proceeding of the National Academy of Science of the USA. 2010; 11. 10.1186/1471-2105-11-129 10.1186/1471-2105-11-129 PMC2984261 20230624

[27] Zuker M, Jacobson AB. Using reliability information to annotate RNA secondary structures. RNA. 1998;4:669–79. 10.1017/s1355838298980116. 9622126 10.1017/s1355838298980116 PMC1369649

[28] Waugh A, Gendron P, Altman R, Brown JW, Case D, Gautheret D, Harvey SC, Leontis N, Westbrook J, Westhof E, Zuker M, Major F. RNAML: a standard syntax for exchanging RNA information. RNA. 2002;8:707–17. 10.1017/s1355838202028017. 12088144 10.1017/s1355838202028017 PMC1370290

[29] Zuker M. Mfold web server for nucleic acid folding and hybridization prediction. Nucleic Acids Res. 2003;31:3406–15. 10.1093/nar/gkg595. 12824337 10.1093/nar/gkg595 PMC169194

[30] Rivas E, Eddy SR. A dynamic programming algorithm for RNA structure prediction including pseudoknots. J Mol Biol. 1999;285:2053–68. 10.1006/jmbi.1998.2436. 9925784 10.1006/jmbi.1998.2436

[31] Ren J, Rastegari B, Condon A, Hoos HH. HotKnots: heuristic prediction of RNA secondary structures including pseudoknots. RNA. 2005;11:1494–504. 10.1261/rna.7284905. 16199760 10.1261/rna.7284905 PMC1370833

[32] Dirks RM, Pierce NA. A partition function algorithm for nucleic acid secondary structure including pseudoknots. J Comput Chem. 2003;24:1664–77. 10.1017/s1355838298980116. 12926009 10.1017/s1355838298980116

[33] Rastegari B, Condon A. Parsing nucleic acid pseudoknotted secondary structure: algorithm and applications. J Comput Biol. 2007;14. 10.1089/cmb.2006.0108 10.1089/cmb.2006.0108 17381343

[34] Sugimoto N, Kierzek R, Turner DH. Sequence dependence for the energetics of dangling ends and terminal base pairs in ribonucleic acid. Biochemisty. 1987;19:4554–8. 10.1021/bi00388a058. 10.1021/bi00388a058 2444250

[35] Zuber J, Sun H, Zhang X, McFayden I, Matthews DH. A sensitivity analysis of RNA folding nearest neighbor parameters identifies a subset of free energy parameters with the greatest impact on RNA secondary structure prediction. Nucleic Acids Res. 2017;45:6168–76. 10.1093/nar/gkx170. 28334976 10.1093/nar/gkx170 PMC5449625

[36] Zuber J, Cabral BJ, McFayden I, Mauger DM, Matthews DH. Analysis of RNA nearest neighbor parameters reveals interdependencies and quantifies the uncertainty in RNA secondary structure prediction. RNA. 2018;24:1568–82. 10.1261/rna.065102.117. 30104207 10.1261/rna.065102.117 PMC6191722

[37] Matthews DH, Disney MD, Childs JL, Schroeder SJ, Zuker M, Turner DH. Incorporating chemical modification constraints into a dynamic programming algorithm for prediction of RNA secondary structure. Proc Nat Acad Sci USA. 2004;101:7287–92. 10.1073/pnas.0401799101. 15123812 10.1073/pnas.0401799101 PMC409911

[38] Andronescu M, Bereg V, Hoos HH, Condon A. RNA STRAND: the RNA secondary structure and statistical analysis database. BMC Bioinformat. 2008;9(1):340. 10.1186/1471-2105-9-340. 10.1186/1471-2105-9-340 PMC2536673 18700982

[39] Turner DH, Matthews DH. NNDB: the nearest neighbor parameter database for predicting stability of nucleic acid secondary structure. Nucleic Acids Res. 2009;38:280–2. 10.1093/nar/gkp892. 10.1093/nar/gkp892 PMC2808915 19880381

[40] Jabbari H, Condon A. A fast and robust iterative algorithm for prediction of RNA pseudoknotted secondary structures. BMC Bioinformatics 2014;15. 10.1186/1471-2105-15-147 10.1186/1471-2105-15-147 PMC4064103 24884954

[41] Gray M, Chester S, Jabbari H. KnotAli: informed energy minimization through the use of evolutionary information. BMC Bioinformat. 2022; 23. 10.1186/s12859-022-04673-3 10.1186/s12859-022-04673-3 PMC9063079 35505276

[42] Fog A. Optimizing Software in C++. (2023). https://www.agner.org/optimize.

